# The Importance of the Prehospital Phase in ST Elevation Myocardial Infarction

**DOI:** 10.5935/abc.20180209

**Published:** 2018-10

**Authors:** Gláucia Maria Moraes de Oliveira, Paolo Blanco Villela

**Affiliations:** Universidade Federal do Rio de Janeiro, Rio de Janeiro, RJ - Brazil

**Keywords:** Cardiovascular Diseases/mortality, Epidemiology, Emergency Medical Services, Healthcare Inequality, Patient Acceptance of Healthcare, Patient Transportation

Notwithstanding the decline which has been observed in recent years, circulatory diseases continue to be the leading cause of death in Brazil.^1^ In spite of recent advances in clinical and interventional treatment,^2^^,^^3^ ischemic heart disease (IHD) was responsible for 116,333 deaths in 2016, of which 80% were due to acute manifestations, principally in the form of acute myocardial infarction (AMI).^4^

In treating AMI patients, especially those with ST-segment elevation, the pre-hospital phase of care plays a crucial role in short- and long-term prognosis. Two data related to this phase deserve our attention. First, we may observe delays in reaching healthcare services. In 80% of cases, these delays last for more than two hours starting from the moment symptoms begin to manifest.^2^^,^^3^ Second, it stands out that 50% of deaths resulting from AMI were recorded precisely during the pre-hospital phase.^2^^,^^3^

Whether these delays in reaching pre-hospital care are predominantly patient-related, including, among other factors, difficulties in recognizing and interpreting symptoms owing to socioeconomic status and/or cultural factors, or whether they are associated with a lack of efficiency within the healthcare system, for example in transporting patients from the place where symptoms onset to the final destination, i.e. the hospital, has yet to be established in the literature.^4^ It is also important to highlight that a recent study indicated that there are sex-specific differences, with greater delays being observed in women, mainly owing to atypical symptoms which lead to delays in the decision to seek healthcare.^5^

Several factors have been associated with delays during the pre-hospital phase, including non-white ethnicity; low socioeconomic status; cultural factors; previous history of angina, diabetes, or hypertension; sociodemographic and situational factors, for example, distance to treatment centers or medical consultation conducted by spouses or relatives; lack of knowledge regarding the meaning of symptoms; anxiety caused by symptoms; access to public and private healthcare systems; the time of day/night when symptoms onset; previous infarction, and associated symptoms, such as profuse sweating, arterial hypotension, and intensity of precordial pain severity.^6^^-^^8^

In Brazil, Rodrigues et al. published a study on predictors of late presentation in 1,297 patients with AMI in a referral center in the country’s South Region, which is able to perform primary angioplasty 24 hours a day, seven days a week.^9^ Approximately 25% (n = 302) of the total patients attended between December 2009 and November 2014 presented a delay of more than six hours, with a significantly higher mortality rate. The independent predictors of late presentation were: black ethnicity, low income level (less than five times minimum wage), and diabetes mellitus. The following variables lost statistical significance after adjusting for multiple logistic regression analysis: female sex, less than eight years of schooling, and occurrence of chronic renal failure. Patients with all of the independent predictors of late presentation took twice as long to reach the hospital as other patients. History of previous heart disease, AMI, or myocardial revascularization were protective factors,^9^ likely owing to the early recognition of a new event and, thus, to reduced delays in seeking medical treatment.

Unfortunately, the authors of this study did not record the distance between the place where symptoms onset and the referral center; they also excluded transfer patients in order to evaluate spontaneous demand.^9^ These two factors also influence mortality related to the pre-hospital phase, even though they are dependent on the healthcare system. On the other hand, they also did not analyze the following confounding factors which are related both to distance between place of symptoms onset and referral center and to transfer patients: ventricular function, time taken to implement mechanical or drug reperfusion therapy, reperfusion therapy success rate, associated procedures, and implementation of adjuvant therapy recommended in the guidelines.^2^^,^^3^

It is also noteworthy that overall mortality differed significantly between the two groups studied, there being no differences observed regarding subgroups, even with major cardiovascular events. This leads us to suppose that other factors that were not analyzed influenced 30-day mortality rate, for instance, mortality related to the performance of highly complex procedures.

One recent study suggests that higher mortality in women due to delays during the pre-hospital phase may be due to the fact that women appear to be more vulnerable to prolonged untreated ischemia.^10^ The longest delays in this study were related to the healthcare system. The authors stress that mortality was even higher in those who arrived at the hospital with more than twelve hours’ delay, as they did not receive any form of reperfusion therapy. In the study carried out by Rodrigues et al., these patients were excluded, thus making it impossible to establish a comparison.^9^

Cultural differences regarding attitudes toward AMI symptoms are also relevant patient-dependent factors. A recent study carried out in Japan showed that patients who were men, were elderly, had lower levels of schooling and had lower self-confidence regarding their understanding of AMI would present delays in seeking medical treatment.^11^ These patient-related factors were also absent from Rodrigues et al.^9^

It is important to highlight that the results of the study carried out by Rodrigues et al. come from a single center whose conditions are quite rare in Brazil, which demonstrates that the continuous availability of mechanical reperfusion therapy was not sufficient to reduce the 30-day AMI mortality rate of about 10% in patients who arrived at the hospital with more than six hours’ delay following onset of symptoms. This is an additional conclusion to the data presented by the authors.

It is necessary to invest not only in the availability of excellent mechanical reperfusion therapy, but also in equal access to healthcare systems, both by improving the population’s socio-economic and cultural conditions and by implementing thrombolytic therapy close to the place where the patient is located during the onset of symptoms or in pre-hospital transport. Only then will we be able to make the mortality rates we observe in our clinical practice match the ones described in the clinical trials of the guidelines.
